# *In Vitro* Studies on the Immunomodulatory Effects of *Pulicaria crispa* Extract on Human THP-1 Monocytes

**DOI:** 10.1155/2020/7574606

**Published:** 2020-09-25

**Authors:** Tarfa Albrahim, Moonerah M. Alnasser, Mashael R. Al-Anazi, Muneera D. ALKahtani, Saad Alkahtani, Ahmed A. Al-Qahtani

**Affiliations:** ^1^College of Health and Rehabilitation Sciences, Department of Health Sciences, Clinical Nutrition, Princess Nourah Bint Abdulrahman University, Riyadh, Saudi Arabia; ^2^Department of Zoology, College of Science, King Saud University, Riyadh, Saudi Arabia; ^3^Department of Infection and Immunity, Research Center, King Faisal Specialist Hospital & Research Center, Riyadh, Saudi Arabia; ^4^Department of Biology, College of Science, Princess Nourah bint Abdulrahman University, Riyadh, Saudi Arabia; ^5^Department of Microbiology and Immunology, Alfaisal University, School of Medicine, Riyadh, Saudi Arabia

## Abstract

**Background:**

*Pulicaria crispa* (*P. crispa*) is a plant from the Compositae family that exhibits antioxidant, anti-inflammatory, antibacterial, and cytotoxic activities.

**Objective:**

The current study aimed at investigating the immunomodulatory effects of *P. crispa* extract in lipopolysaccharide- (LPS-) stimulated human monocytic THP-1 cells.

**Methods:**

To induce macrophage differentiation, THP-1 cell lines were treated with phorbol-12-myristate 13-acetate, followed by exposure to LPS with or without 50 or 100 *μ*g/ml of *P. crispa* extract. The following tests were employed to test the immunomodulatory effects of the extract: MTT assay, ELISA, Western blotting analysis, cell migration and phagocytosis assays, and Annexin V staining method.

**Results:**

Exposure to 100 *μ*g/ml *P. crispa* extract significantly reduced THP-1 cell proliferation, migration, and phagocytosis (in LPS-stimulated cells, but not in unstimulated cells). Moreover, the extract alone significantly reduced the rate of THP-1 cell apoptosis, while it increased the rate of late apoptosis. Molecular investigations showed that treatment with *P. crispa* extract significantly upregulated the expression of ERK1, p-MAPK, P-P38, and Bcl2, while it significantly reduced the expression of ERK5, Bax, NF-*κ*B, P-NF-*κ*B, CCL1, CCL2, CCL5, CCL22, CXCL1, and CXCL10.

**Conclusion:**

*Pulicaria crispa* extract exhibited anti-inflammatory, antiproliferative, antimigratory, and antiphagocytic effects in LPS-stimulated THP-1 cells. Future studies should investigate these mechanisms in animal models with chronic inflammatory diseases.

## 1. Introduction

Macrophages (M*Φ*s) are cells of the innate immune system that start differentiating in the human body in the second trimester of gestation. They are essential in maintaining immune homoeostasis and play several roles in initiating and regulating the immune responses to foreign antigens [1]. Generally, M*Φ*s were seen primarily as phagocytes that engulf bacteria and dying cells [2]; however, substantial recent evidence uncovered other functions executed by various surface and intracellular receptors of these cells. Such functions include regulation of bone remodeling [3], erythropoiesis [4], brain development [5], iron recycling [6], and tissue regeneration [7]. M*Φ*s are classically divided into M1 and M2 cells: M1 M*Φ*s which exhibit proinflammatory, bactericidal, and phagocytic activities and M2 cells which are involved in tissue regeneration and regulating the immune response via interleukin- (IL-) 10 secretion [8].

The role that M*Φ*s play in inflammation is multifaceted [1]. Exposure to damage-associated molecular patterns (DAMPs), released from damaged cells, causes M*Φ*s to secrete several cytokines including IL-1*β*, IL-6, tumor necrosis factor-*α*, and proinflammatory eicosanoids. These secreted molecules cause vasodilatation and edema, followed by neutrophil recruitment [9]. Further, following phagocytosis and lysis of foreign organisms, M*Φ*s present antigens from its surface major histocompatibility complex- (MHC-) II receptor molecules to allow T-helper cells to initiate adaptive immune response [10].

During resolution of the inflammation, some M*Φ*s undergo apoptosis or convert from an initial proinflammatory (M1) phenotype to a healing proresolving (M2) phenotype consistent with plasticity of myeloid cells [11]. Examples of this behavior were observed in the muscles [12] and kidneys [13]. However, M*Φ*s sometimes escape these destinies and become more active and with a longer lifespan [14]. The latter cells have been implicated in chronic inflammatory and autoimmune diseases [15].

*Pulicaria crispa* (also known as *Francoeuria crispa*) is a plant from the Compositae family that commonly grows in the Middle East countries, including Saudi Arabia and Egypt. Although it has been used in traditional medicine for a long time, it has become a topic of interest for medicinal research since the 1980s [16]. Previous studies have shown that *P. crispa* derivatives exert antioxidative [17], anti-inflammatory [18], and chemopreventive activities [19]. A phytochemical screening of *P. crispa* extract attributed these effects to the presence of coumarins, tannins, and flavonoids [18, 20].

The extracts of *P. crispa* have shown anti-inflammatory, as well as immunostimulatory effects. For example, several studies have shown antimicrobial effects for *P. crispa* extracts against Gram-negative bacteria [21–23], *Mycobacteria* [20, 21], *Candida albicans* [17], *Schistosoma mansoni* [24], *Leishmania* [25], and hepatitis B virus [26]. On the other hand, extracts from the *Pulicaria* species have been shown to inhibit neutrophil infiltration and alleviate oxidative stress. It was also postulated that it suppresses the generation of nitric oxide [27] and modulates the expression of intracellular adhesion molecule-1 (ICAM-1), tumor necrosis factor- (TNF-) *α* [18], and prostaglandin E2 [18]. Therefore, further study of their effects on the immune system, especially at the molecular level, will be particularly insightful.

Despite the benefits of the immune inflammatory response to foreign antigens, a dysregulated immune response can lead to a wide array of chronic inflammatory conditions [28]. Several *in vivo* and *in vitro* models have been proposed to study the anti-inflammatory and immunomodulatory effects of various phytochemicals and pharmaceutical compounds. Of these models, human leukemic THP-1 monocytes have attracted attention as a valid *in vitro* model to investigate the molecular mechanisms of inflammation [29–31]. For example, previous studies have used this model to evaluate the anti-inflammatory effects of several phytochemicals extracted from different organisms, including *Corydalis crispa*, *Corydalis dubia*, *Ajania nubigena*, *Meconopsis simplicifolia*, *Ocimum sanctum*, and *Uncaria tomentosa* [32–34].

Owing to the scarcity of molecular research regarding the immunomodulatory effects of *P. crispa* effects, the current study was performed to utilize human THP-1 cells as an *in vitro* model to test the effects of their exposure to lipopolysaccharide (LPS) and *P. crispa* extract.

## 2. Materials and Methods

### 2.1. *P. crispa* Extract

The preparation of the extract was described in detail elsewhere [26].

### 2.2. Growth and Maintenance of THP-1 Cells

The human monocytic THP-1 cells were cultured in RPMI media supplemented with 10% fetal bovine serum (FBS), 100 IU/ml penicillin, 100 *μ*g/ml streptomycin, and 3.7 g/l sodium bicarbonate. The cells were maintained in 37°C temperature and 5% CO_2_.

### 2.3. Differentiation of THP-1 Cells into Macrophage-Like Cells

THP-1 cells (5 × 10^5^) were incubated with 100 ng/ml of phorbol 12-myristate 13-acetate for 48 hours. The cells were then washed with RPMI 1640 serum-free medium to eliminate undifferentiated cells.

### 2.4. Treatment of M*Φ*s with LPS and/or *P. crispa* Extract

Differentiated cells were treated with 100 ng/ml LPS, isolated from *Escherichia coli* O26:B6 bacteria (Sigma-Aldrich, St. Louis, MO, USA), alone, LPS + 50 *μ*g/ml or LPS + 100 *μ*g/ml of *P. crispa* extract. In some experiments, differentiated cells were treated with the extract alone (50 or 100 *μ*g/ml). The duration of the treatment was either 4 hours or 6 hours.

### 2.5. MTT Cell Proliferation Assay

The MTT assay kit (Abcam, Cambridge, MA, USA) was used following the manufacturer's instructions. The treated cells, at a density of 5 × 10^4^ cells/well, were incubated with either 50 or 100 *μ*g/ml *P. crispa* extract for 1 to 8 days. After incubation with MTT reagent for three hours, the absorbance was read in the SpectraMax i3x Multi-Mode Microplate Reader (Molecular Devices, Sunnyvale, CA, USA) at 540 nm wavelength.

### 2.6. Expression Analysis by Real-Time Quantitative Reverse-Transcriptase Polymerase Chain Reaction (qRT-PCR)

Total RNA was extracted from treated THP-1 cells using the QIAamp RNA Blood Mini Kit (Qiagen, Hilden, Germany) following the manufacturer's instructions, followed by a double-stranded cDNA synthesis using All-in-One cDNA Synthesis SuperMix (Biotool, Houston, TX, USA). The generated cDNA was used for real-time PCR experiments using target-specific primers and probes purchased from Applied Biosystems (Foster City, CA, USA). The mRNA expression levels of studied genes were normalized to GAPDH.

### 2.7. Enzyme-Linked Immunosorbent Assay (ELISA)

Supernatants from treated cells were collected for quantitation of secreted proteins. The DuoSet® ELISA kit (R&D Systems, Minneapolis, MN, USA) was used to determine the quantities of IL-1*β*, IL-8, CCL22, and CXCL10 following the manufacturer's instructions. Briefly, supernatants were added to wells precoated with protein-specific antibodies, incubated for 2 hours, washed three times with PBS, and incubated with Streptavidin-horseradish peroxidase (HRP) for 20 minutes. After washing, tetramethylbenzidine (*TMB*) substrate was added for 20 minutes, the reaction was stopped, and the color intensity was measured at wavelength 450 nm.

### 2.8. Western Blotting Analysis

The expression of ERK5, Bax, Bcl2, Cyclin-D1, ERK1, MEK1, NF-*κ*B, P-IKB-*α*, P-MAPK, P-NF-*κ*B, and P-P38 in THP-1 cells was evaluated using the Western blotting analysis. The treated cells were lysed in the RIPA buffer, and the cellular proteins were separated on 12% SDS-polyacrylamide gel electrophoresis (PAGE). The proteins were then transferred to polyvinylidene difluoride (PVDF) membranes which were treated with 5% nonfat dry milk. The membranes were then probed with protein-specific primary antibodies overnight at 4°C, washed, and incubated with HRP-conjugated secondary antibodies. The reaction was detected by the addition of SuperSignal West Pico 16 Chemiluminescent substrate (Thermo Fisher Scientific, Waltham, MA). The bands were visualized on a GE Amersham Imager 600, and the proteins were quantified using the ImageJ software (National Institutes of Health, Bethesda, MD).

### 2.9. Cell Migration Assay

Cell migration was evaluated using the CytoSelect™ 24-Well Cell Migration Assay (Cell Biolabs, San Diego, CA, USA) following manufacturer's instructions. Briefly, treated cells were incubated in a serum-free RPMI in the upper chamber. The cells were allowed to migrate to the lower chamber filled with RPMI and FBS. The chamber was then incubated for 24 hours, and the migration capability was analyzed by reading the fluorescence of the GR dye at a wavelength of excitation/emission 480/520 nm.

### 2.10. Phagocytosis Assay

Treated THP-1 cells were incubated with Alexa Fluor 405-labeled Zymosan particles Molecular Probes (Carlsbad, CA, USA) for 1 hour in a serum-free RPMI medium at 37°C. Cells were washed three times with PBS to eliminate noninternalized fluorescent beads. The cells were analyzed by measuring the absorbance at OD of 405 nm.

### 2.11. Apoptosis Assay

Apoptosis was measured in treated cells by flow cytometry using Annexin V/propidium iodide double-staining as described before [35, 36], and the analysis was performed in FACScan equipped with the Cell Quest software (Becton Dickinson, Cockeysville, MD, USA).

### 2.12. Statistical Analysis

Data generated from the aforementioned assays were expressed as mean ± standard error of mean and were presented in bar graphs with error bars. To compare the groups in, *t* test was used. A *p* value was considered significant if less than 0.05.

## 3. Results

### 3.1. The Effects of *Pulicaria crispa* Extract on THP-1 Cell Proliferation

**Figure 1:**
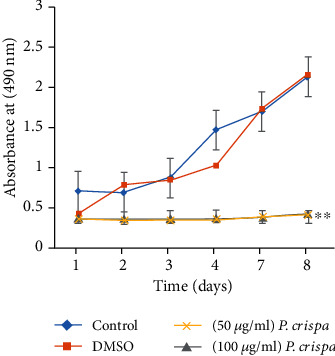
The effect of *P. crispa* extract on proliferation of THP-1 cells. The *y*-axis shows absorbance at 490 nm after incubation of cells treated with DMSO alone, 50 *μ*g/ml or 100 *μ*g/ml of *P. crispa* extract relative to untreated cells (control). ∗∗*p* value < 0.01 (treatment vs. control). Values represent the mean of three different experiments done in triplicate ± SEM for each time point tested. Statistical analysis was performed using the *t* test.

To assess the effects of *P. crispa* extract on THP-1 cell proliferation, MTT assay was used by incubating the cells with 50 and 100 *μ*g/ml of the extract for 8 days. Analysis of the change in proliferation of the cells showed that while the vehicle control (DMSO) did not produce any significant difference in THP-1 cell proliferation in comparison to control monocytes, both concentrations of *P. crispa* extract significantly reduced the proliferation of the cells at each time point (*p* = 0.01) (Figure 1).

### 3.2. The Effects of *P. crispa* Extract on mRNA Expression in THP-1 Cells

**Figure 2:**
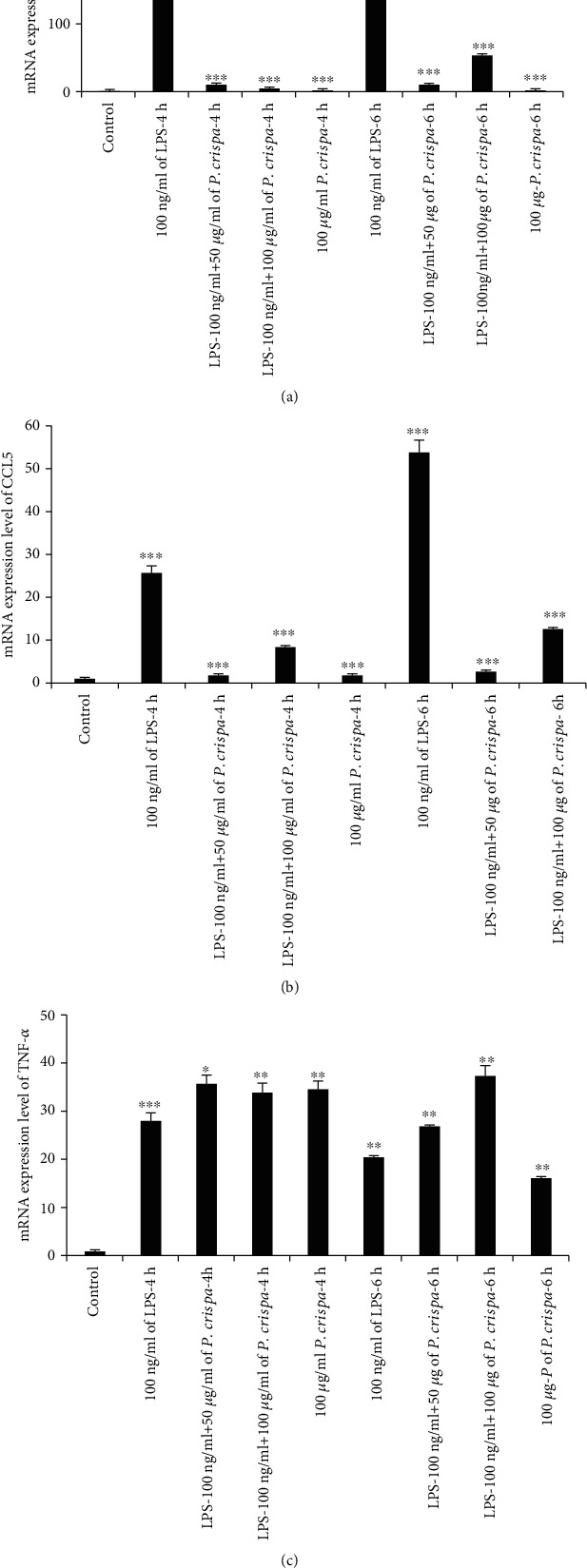
qRT-PCR analysis for the expression of (a) CCL2, (b) CCL5, and (c) TNF-*α*. The mRNA expression levels were normalized with GAPDH. Control cells were compared with LPS-treated cells. All other cells with different treatments were compared with LPS-treated cells. The data were expressed as mean ± standard error done in triplicate for three independent experiments. Statistical significance was established using the *t* test. ∗<0.05; ∗∗<0.01; ∗∗∗<0.001.

To evaluate the mRNA expression of CCL2, CCL5, and TNF-*α* in THP-1 cells exposed to LPS or different concentrations of *P. crispa* extract, we used the qRT-PCR analysis. Exposure of cells to 100 ng/ml LPS at 4 and 6 hours significantly increased mRNA expression of CCL2 and CCL5 in a time-dependent manner (*p* = 0.001). However, treatment of LPS-exposed cells with *P. crispa* extract at 50 and 100 *μ*g/ml was associated with a significant decrease in CCL2 and CCL5 expression in comparison to LPS alone (*p* = 0.001) (Figures 2(a) and 2(b)). On the other hand, treatment of THP-1 cells with *P. crispa* extract alone resulted in a significant increase in mRNA expression of TNF-*α* at 4 and 6 hours (*p* ≤ 0.01) (Figure 2(c)).

### 3.3. Protein Array Expression Analysis

**Figure 3:**
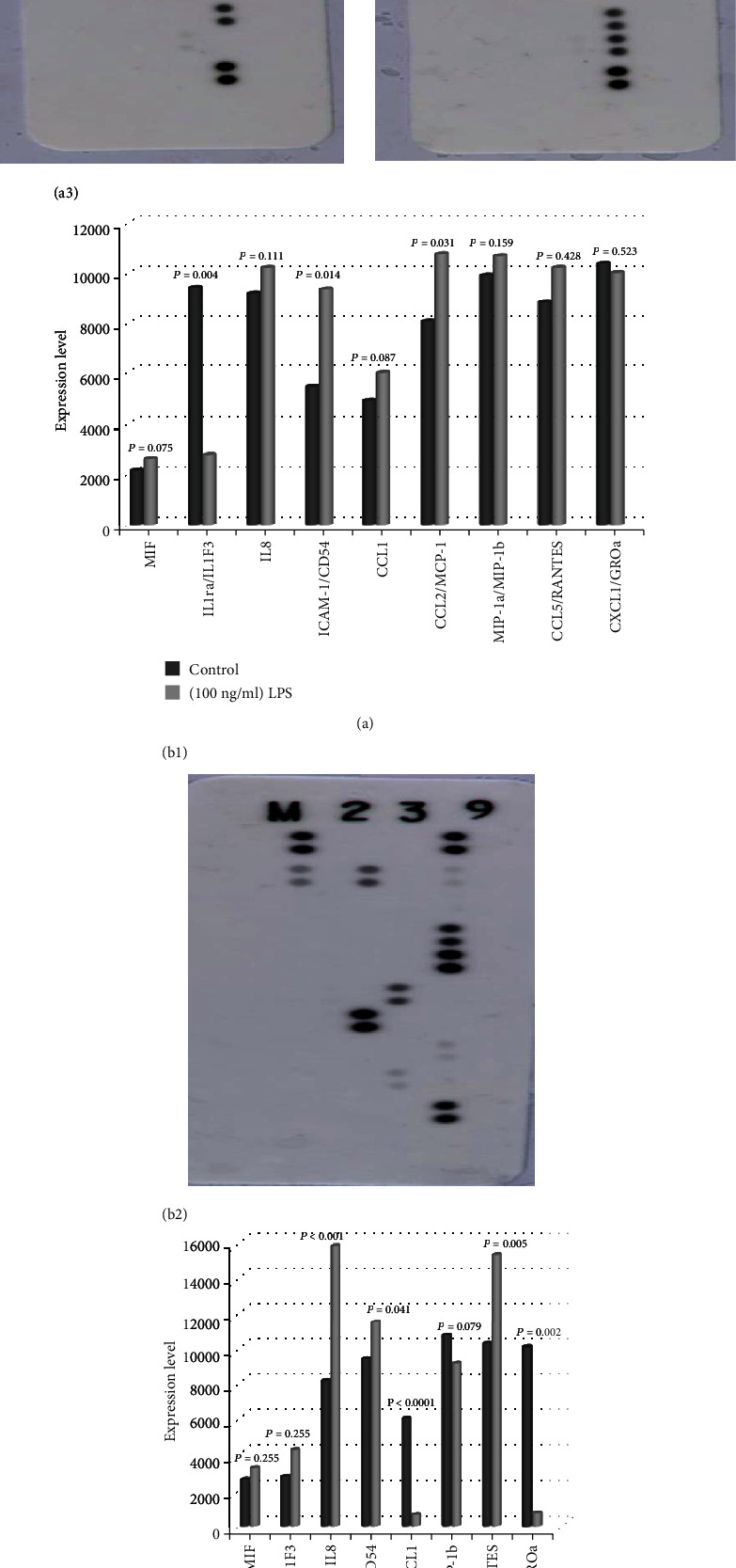
Protein expression array analysis. (a) Control THP-1 cells (a1); cells treated with (100 ng/ml) of LPS (a2); histograms comparing the protein array expression results from controls and THP-1 cells treated 100 ng/ml of LPS (a3). (b) Cells treated with B2 B1 25 (100 ng/ml) of LPS + 100 *μ*g/ml of *P. crispa* (b1); graphical representation of LPS-treated cells (shown in a2) compared with cells treated with LPS + *P. crispa* extract (b2). Statistical significance was established using the *t* test.

Protein array was used to simultaneously detect expression of several proteins in treated cells. Comparison of control cells to cells treated with 100 ng/ml LPS showed significantly higher expression of ICAM1/CD54 (*p* = 0.01) and CCL2/MCP1 (*p* = 0.03), as well as significantly lower expression of IL1ra/IL1F3 (*p* = 0.004). However, no statistically significant differences were observed with regard to MIF, IL8, CCL1, MIP1a/MIP1b, CCL5/RANTES, and CXCL1/GROa (Figure 3(a)). In contrast, LPS-exposed cells treated with 100 *μ*g/ml *P. crispa* extract showed significant reductions with regard to CCL1 and CXCL1/GROa expression (*p* < 0.05). In addition, a significant increase in CCL5/RANTES, ICAM-1/CD54, and IL8 was observed. No significant differences were detected in the expression of MIF, IL1ra/IL1F3, and MIP-1a/MIP-1b (Figure 3(b)).

### 3.4. ELISA Assay Results

**Figure 4:**
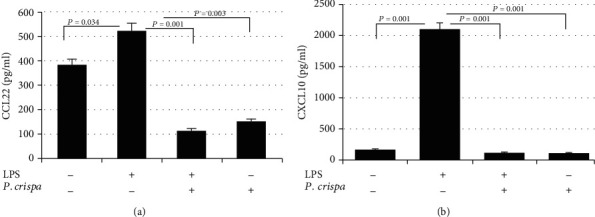
ELISA analysis of THP-1 cells after stimulation with LPS (100 ng/ml) with or without *P. crispa* extract (100 *μ*g/ml). The expressions of CCL22 (a) and CXCL10 (b) are detected. Culture supernatants were harvested 6 hours after stimulation and processed for ELISA. All samples were processed in triplicate for three independent experiments ± SEM. Statistical significance was established using the *t* test.

ELISA assay was used to confirm the results obtained by the protein array analysis. The supernatants of cultured cells were harvested after six hours of stimulation. The analysis of cells exposed to LPS only showed significant increase in the production of CCL22 (*p* = 0.03) and CXCL10 (*p* = 0.001) in comparison to control cells. On the other hand, LPS-exposed cells treated with *P. crispa* extract showed significant reduction in the production of CCL22 and CXCL10 (*p* = 0.001) (Figures 4(a) and 4(b)).

### 3.5. Western Blotting Analysis

**Figure 5:**
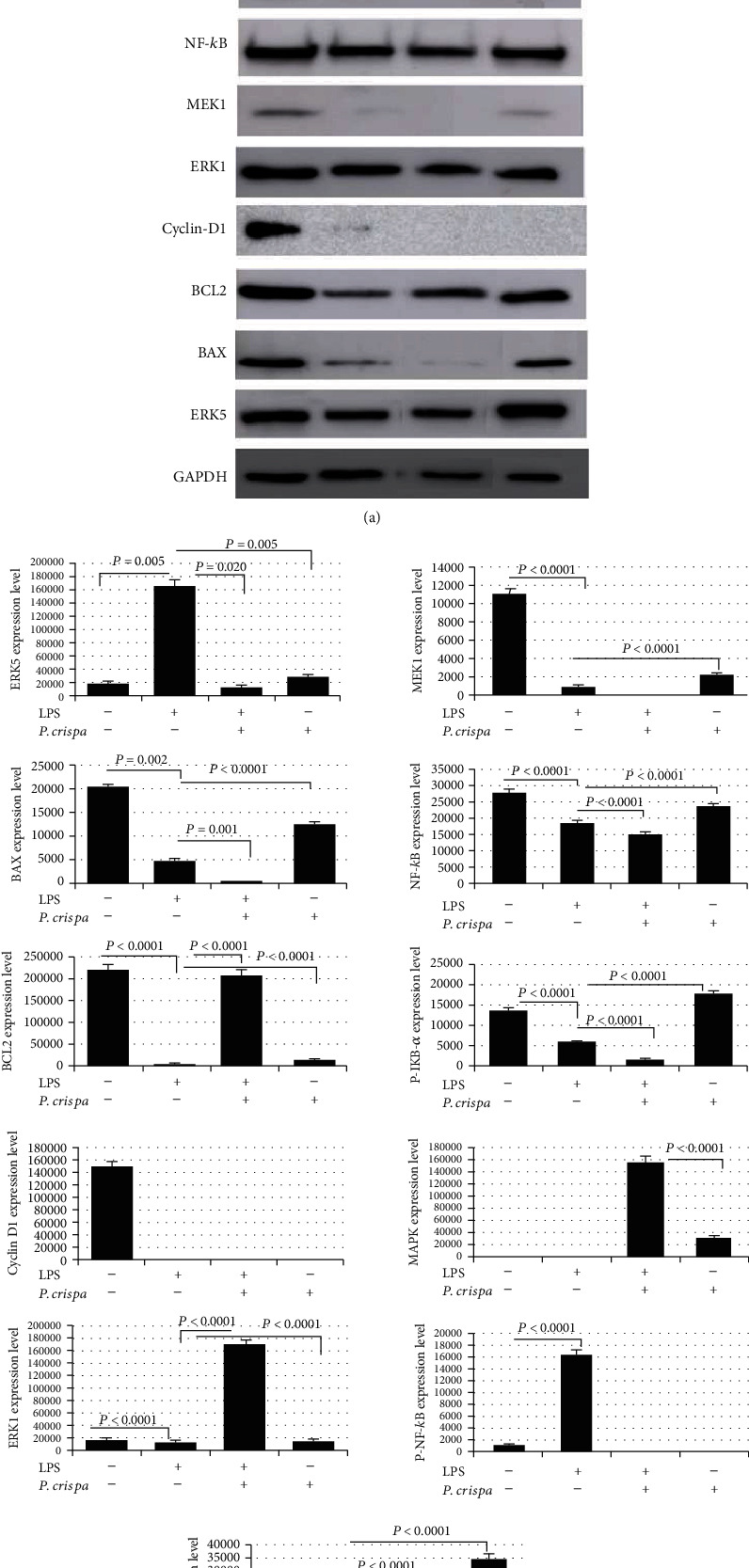
Expression analysis of proteins in THP-1 cells. (a) Western blot analysis and (b) densitometric analysis of the expression of ERK5, BAX, BCL2, Cyclin-D1, ERK1, MEK1, NF-*κ*B, P-IKB-*α*, P-MAPK, P-NF-*κ*B, and P-P38 proteins. Cells were either untreated (control), treated with 100 ng/ml of LPS, treated with 100 ng/ml of LPS + 100 *μ*g/ml of *P. crispa* extract, or treated with 100 *μ*g/ml of *P. crispa* extract alone. All data are expressed as mean ± SEM. All samples were processed in triplicate in three independent experiments. Statistical significance was established using the *t* test.

The expression of 11 cell survival, apoptosis, and inflammatory proteins was measured through Western blotting analysis. In comparison to nontreated cells, LPS-exposed cells showed significant overexpression of ERK5, p-NF-*κ*B, and p-P38, as well as significant decreased expression of Bcl2, Bax, MEK1, ERK1, P-IKB-*α*, and NF-*κ*B. However, the expression of p-MAPK and Cyclin-D1 could not be detected in LPS-exposed cells (Figure 5). In contrast, LPS-exposed cells treated with 100 *μ*g/ml *P. crispa* extract showed significant downregulation of ERK5, Bax, P-IKB-*α*, and NF-*κ*B, as well as upregulation of Bcl2, ERK1, p-MAPK, and p-P38 in comparison to LPS only-treated cells. Of note, the expression of MEK1, P-NF-*κ*B, and Cyclin-D1 could not be detected in cells treated with LPS + *P. crispa* extract (Figure 5).

### 3.6. Cell Migration Assay

**Figure 6:**
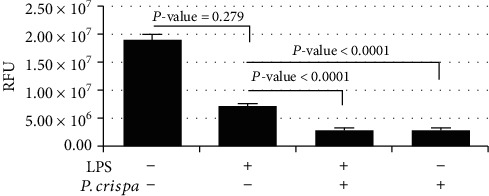
Migratory potential of THP-1 cells. Cells were treated with LPS (100 ng/ml) in the presence or absence of *P. crispa* extract (100 *μ*g/ml). Migration capability of cells was quantified by cell migration assay. Values represent the mean of triplicate experiments done in triplicate for each time point tested. Statistical significance was established using the *t* test. RFU: relative fluorescence units.

To assess the effect of *P. crispa* extract on the ability of THP-1 cell to respond to external stimuli, cell migration assay was used. The analysis showed that LPS-exposed cells were not significantly different from untreated control cells (*p* = 0.279). However, cells treated with *P. crispa* alone or in combination with LPS showed significantly reduced (*p* < 0.0001) cell migration capability in comparison to cells treated with LPS alone (Figure 6).

### 3.7. Phagocytosis Assay

**Figure 7:**
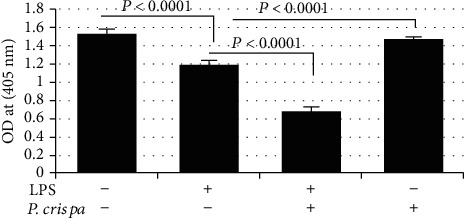
Phagocytosis assay using Zymosan particles. THP-1 cells were treated with LPS (100 ng/ml) and/or *P. crispa* extract (100 *μ*g/ml). Values represent the mean of triplicate experiments done in triplicate for each time point tested. Statistical significance was established using the *t* test

The results of the phagocytosis assay showed that LPS-exposed THP-1 cells exhibited significantly less phagocytic activity than control untreated cells (*p* < 0.0001). Further, the addition of *P. crispa* extract to LPS-exposed cells reduced their phagocytic activity (*p* < 0.0001). However, THP-1 cells treated with *P. crispa* extract alone exhibited significantly higher phagocytic activity than cells treated with LPS alone (*p* < 0.0001) (Figure 7).

### 3.8. Flow Cytometry-Based Detection of Apoptosis

**Figure 8:**
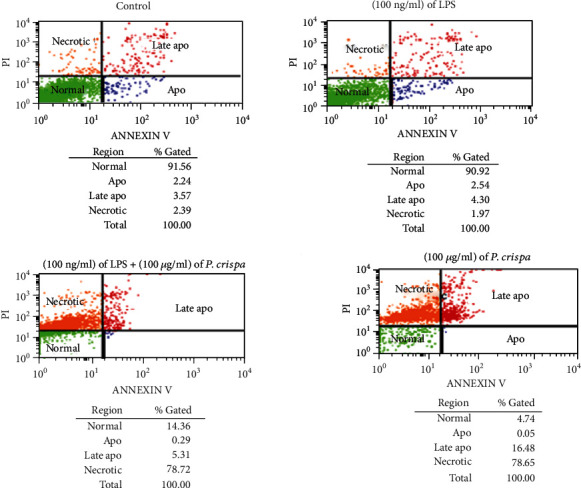
Flow cytometry using Annexin V staining method to analyze apoptosis in THP-1 cells. Analysis of apoptosis in the cells exposed to 100 ng/ml LPS and/or 100 *μ*g/ml *P. crispa* extract. Cells that were propidium iodide (PI) negative and Annexin V negative are considered healthy, cells, PI negative and Annexin V positive cells are considered apoptotic, and cells that are positive to both PI and Annexin V considered necrotic. ∗ indicates significance.

This assay was conducted using Annexin V/PI double-staining method. As shown in Figure 8 and Table 1, the majority (>90%) of monocyte cells and LPS-treated THP-1 cells remained viable with no statistically significant difference in both groups with regard to apoptotic and late apoptotic cells (*p* = 0.77). However, exposure of cells to *P. crispa* extract significantly decreased the number of apoptotic cells and increased the number of late apoptotic cells in comparison to control cells (*p* = 015). In comparison to LPS-treated cells, THP-1 cells treated with both LPS and *P. crispa* extract did not show significant differences in the percentage of apoptotic and late apoptotic cells (*p* = 0.77).

## 4. Discussion

Persistent activation of M*Φ*s has been implicated in several chronic inflammatory and autoimmune diseases [37]. M*Φ*s under prolonged stimulation secrete large quantities of proinflammatory and chemotactic molecules. These cells also become more resistant to apoptosis [38]. Here, we used an *in vitro* model (THP-1 human monocytes) to simulate activated macrophages during an inflammatory response.

To discuss our results, some physiological perspective is needed to be considered. Activated M*Φ*s usually exhibit increased expression of molecules involved in proinflammatory signals such as NF-*κ*B. This protein is essential in the pathway that increases the production of some interleukins, such as IL-1, IL6, and TNF-*α* [39]. Furthermore, activated macrophages orchestrate the immune response by recruiting other cells into the inflammation area through chemotactic proteins, including CCL1, CCL2, CC5, CCL22, CXCL1, and CXCL10 [40]. Inside activated macrophages, the transcriptional program shifts in favor of prolonged survival, increased proliferation, and enhanced migration [41]. In this study, we further investigated these mechanisms and evaluated the role of *P. crispa* extract in modulating these functions in M*Φ*s.

Several studies have documented that biochemical constituents, such as Alkaloid, Flavonoids, and Terpenoids, in the extract of *P. crispa* might be associated with antimicrobial and anti-inflammatory activity [20, 22]. Another study found that organic constituents from *P. undulata* significantly promoted apoptosis in hepatocellular carcinoma (HCC) HepG2 cells and elevated the expression of miR-34a. Furthermore, the extract enhanced the caspase 3/9 and proapoptotic p53 protein expression with plummeting of B-cell lymphoma-2 protein expression. Therefore, the antitumor activity of *P. crispa* through regulation of p53/B-cell lymphoma-2/caspases signaling pathway in the HCC HepG2 cells by overexpressing miR-34a is suggested. Such an antitumor effect might be attributed to the presence of triterpenoids and coumarins [42]. Also, Foudah et al. reported noticeable antioxidant and antimicrobial properties, and this could be owing to the presence of flavonoids, phenols, and tannins in the methanol extract of *P. crispa* plant [17].

Overall, this study showed that treatment of LPS-stimulated cells with 50 and 100 *μ*g/ml of *P. crispa* extract inhibited THP-1 cell proliferation, migration, and phagocytosis and altered the expression of several inflammatory- and cell survival/apoptosis-related proteins. To our knowledge, this is the first *in vitro* study to evaluate the immunomodulatory and anti-inflammatory effects of *P. crispa* in LPS-stimulated human THP-1 cells.

First, results from the MTT assay showed that both concentrations of *P. crispa* extract inhibited THP-1 cell proliferation. In parallel, downregulation of ERK5 and lack of MEK1 and Cyclin-D expression were observed in treated cells. ERK5 is a member of the MAPK family that responds to extracellular stimuli, such as stress and growth factors, to regulate cell proliferation and differentiation via its downstream targets including AKT serine-threonine protein kinase and myocyte enhancer factor [43]. Downregulation of this MAPK protein may explain the observed proliferation inhibition. In addition, the lack of MEK1, another MAPK protein involved in cell growth and proliferation, [44] and Cyclin-D (a cell cycle regulator [45] expression in *P. crispa*-exposed cells could explain the ability of the extract's constituents to suppress the intracellular proliferation signaling pathways. However, flow cytometry plots showed a large increase in necrotic cells following treatment with extract, which is also a sign of potential toxicity of *P. crispa* extract.

Interestingly, we also observed the increased expression of prosurvival (ERK1, MAPK, and P-P38) and antiapoptotic (Bcl2) proteins in THP-1 cells, exposed to *P. crispa* extracts. Activated ERK-1 activates the MAPK signal transduction pathway, which regulates cell cycle progression and survival [46]. Moreover, previous studies have shown that phosphorylated-p38 (P-P38) expression is linked to enhanced cell survival in normal and malignant cells [47, 48]. In parallel, the Annexin V staining method showed that exposure to *P. crispa* extract alone reduces the frequency of THP-1 cell apoptosis yet increases the frequency of late apoptosis. This discrepancy may be caused by a shift in the expression pattern after exposure to the *P. crispa* extract.

Another interesting finding was the ability of *P. crispa* extract to inhibit the migration of stimulated THP-1 cells. Molecular investigations revealed supporting results, i.e., treatment with *P. crispa* extract significantly downregulated the expression of CCL1, CCL2, CCL5, CCL22, CXCL1, and CXCL10 (all are known chemoattractant proteins) [49] in human THP-1 cells. These chemokines are produced by macrophages, and their function is to recruit leukocytes to the inflammation site. Of interest, it has been shown that *P. crispa* extract ameliorates leucocytes infiltration [27]; however, the current study is the first to focus on the molecular mechanisms underlying these cellular changes. No significant effects were observed with regard to ICAM1 expression upon *P. crispa* extract exposure, which may indicate that the extract constituents may inhibit the cellular migration, but their effects on leukocyte adhesion and cell-to-cell interactions are not significant [50].

In addition, the *P. crispa* extract showed multiple anti-inflammatory effects in the present study. First, the significant reduction in NF-*κ*B and P-NF-*κ*B expression in LPS-stimulated cells occurred after treatment with 100 *μ*g/ml of the extract. Pereira and colleagues showed that inhibiting ERK5 suppresses the NF-*κ*B signaling pathway [51]. Another study by Kloster et al. reported that MEK signaling is important for activating NF-*κ*B signaling [52]. The observation that MEK expression was absent in LPS-stimulated cells after *P. crispa* extract exposure may explain the observed reduction in NF-*κ*B signaling. Moreover, it has been shown that activation of NF-*κ*B mainly occurs via IKB kinase-mediated phosphorylation of the inhibitory molecule IKB-*α* [53]. The downregulated NF-*κ*B expression may explain the observed underexpression of chemotactic factors in the current study [54]. However, to our surprise, *P. crispa* extract increased TNF-*α* expression in LPS-stimulated and unstimulated cells. Although this would need confirmation in future studies, it may indicate that *P. crispa* extract constituents target other regulatory factors that control the TNF-*α* expression.

Phagocytosis is a major function of macrophages in the innate immune response against foreign bodies. It is regulated by several cytokines including TNF-*α* and IL-1 [55]. However, it may be implicated in several chronic inflammatory diseases such as chronic infections, systemic lupus erythematosus, and autoimmune anemia [56]. The present study showed that *P. crispa* extract inhibited the phagocytic activity in LPS-stimulated cells. However, surprisingly, it enhanced phagocytosis in unstimulated THP-1 cells. This differential effect needs to be further confirmed in future studies.

In conclusion, treatment of THP-1 cells with *P. crispa* extract significantly reduced cell migration, proliferation, and phagocytosis in LPS-stimulated cells. Moreover, it significantly reduced the expression of various chemotactic and cell survival-related proteins. These results highlight the anti-inflammatory and immunomodulatory effects of *P. crispa* extract in LPS-stimulated THP-1 cells. Future studies should investigate these mechanisms in animal models with chronic inflammatory diseases.

## Figures and Tables

**Table 1:** Flow cytometry using Annexin V staining method to analyze apoptosis in THP-1 cells.

	Control	(100 ng/ml) of LPS	*p* value
Apoptosis	2.24	2.54	0.774
Late apoptosis	3.57	4.3	
	(100 ng/ml) of LPS	(100 ng/ml) of LPS + (100 *μ*g/ml) of *P. crispa* extract	*p* value
Apoptosis	2.54	0.29	0.774
Late apoptosis	4.3	5.31	
	Control	(100 *μ*g/ml) of *P. crispa* extract	*p* value
Apoptosis	2.24	0.05	0.015
Late apoptosis	3.57	16.48	

## Data Availability

The data used to support the findings of this study are available from the corresponding author upon request.

## Abbreviations

CCL: C-C motif chemokine ligand
CXCL: C-X-C motif ligand 2
ERK: Extracellular-signal regulated kinase
ICAM: Intracellular adhesion molecule
IKB-*α*: Inhibitor kappa B-*α*
LPS: Lipopolysaccharide,
MAPK: Mitogen activated protein kinase
TNF: Tumor necrosis factor
M*Φ*s: Macrophages
IL: Interleukin
DAMPS: Damaged associated molecular patterns
MHC: Major histocompatibility complex
ICAM-1: Intracellular adhesion molecule
FBS: Fetal bovine serum
qRT-PCR: Quantitative reverse-transcriptase polymerase chain reaction
ELISA: Enzyme-linked immunosorbent assay
TMB: Tetramethylbenzidine
HRP: Streptavidin-horseradish peroxidase
PVDF: Polyvinylidene difluoride
HCC: Hepatocellular carcinoma.
